# V2X Communication between Connected and Automated Vehicles (CAVs) and Unmanned Aerial Vehicles (UAVs)

**DOI:** 10.3390/s22228941

**Published:** 2022-11-18

**Authors:** Ozgenur Kavas-Torris, Sukru Yaren Gelbal, Mustafa Ridvan Cantas, Bilin Aksun Guvenc, Levent Guvenc

**Affiliations:** Automated Driving Lab, Ohio State University, 1320 Kinnear Rd, Columbus, OH 43212, USA

**Keywords:** connected and automated vehicles, unmanned aerial vehicles, vehicle to everything (V2X) communication, DSRC communication, 4G communication

## Abstract

Connectivity between ground vehicles can be utilized and expanded to include aerial vehicles for coordinated missions. Using Vehicle-to-Everything (V2X) communication technologies, a communication link can be established between Connected and Autonomous vehicles (CAVs) and Unmanned Aerial vehicles (UAVs). Hardware implementation and testing of a ground-to-air communication link are crucial for real-life applications. In this paper, the V2X communication and coordinated mission of a CAV & UAV are presented. Four methods were utilized to establish communication between the hardware and software components, namely Dedicated Short Range communication (DSRC), User Datagram Protocol (UDP), 4G internet-based WebSocket and Transmission Control Protocol (TCP). These communication links were used together for a real-life use case scenario called Quick Clear demonstration. In this scenario, the first aim was to send the accident location information from the CAV to the UAV through DSRC communication. On the UAV side, the wired connection between the DSRC modem and Raspberry Pi companion computer was established through UDP to get the accident location from CAV to the companion computer. Raspberry Pi first connected to a traffic contingency management system (CMP) through TCP to send CAV and UAV location, as well as the accident location, information to the CMP. Raspberry Pi also utilized WebSocket communication to connect to a web server to send photos that were taken by the camera that was mounted on the UAV. The Quick Clear demonstration scenario was tested for both a stationary test and dynamic flight cases. The latency results show satisfactory performance in the data transfer speed between test components with UDP having the least latency. The package drop percentage analysis shows that the DSRC communication showed the best performance among the four methods studied here. All in all, the outcome of this experimentation study shows that this communication structure can be utilized for real-life scenarios for successful implementation.

## 1. Introduction

Connectivity between ground vehicles has accelerated the research and development of ground vehicle-based intelligent transportation systems and applications. Connected and Autonomous vehicles (CAVs) can communicate with roadway infrastructure around them through Vehicle-to-Infrastructure (V2I) communication, which brings about traffic light signal and timing (SPaT) based vehicle speed planning to save fuel and improve mobility. CAVs are also able to communicate with each other through Vehicle-to-Vehicle (V2V) communication. Through V2V communication, CAVs can share their position, speed, and acceleration information with other CAVs around them. Using the nearby vehicle information, CAVs can execute coordinated moves such as forming platoons and convoys to save fuel. CAVs can also communicate with other traffic agents such as pedestrians through Vehicle-to-Everything (V2X) communication. Using V2X, CAVs can be utilized in preventing unwanted collisions and accidents with pedestrians and bicyclists, as well as improving overall safety for other ground transportation agents.

There are different protocols that can be utilized when it comes to V2X communication. The most commonly used communication protocols in the automotive industry for connected vehicle (CV) applications to share data between various transportation agents are Dedicated Short Range Communication (DSRC), 5G and 4G-LTE communication. Future 6G usage will be similar to 5G and 4G-LTE communication, but it will be much faster.

DSRC, also known as IEEE802.11p, is a Wireless Local Area Network (WLAN) protocol with a dedicated bandwidth of 75 MHz in the 5.850 GHz to 5.925 GHz band that has been allocated by the US Federal Communication Commission [1]. DSRC has been used for communication needs in the automotive industry and has been employed successfully for a wide variety of V2I, V2V and V2X applications. DSRC-based systems are also cheap and easy to implement since the required technology has been developed to a good extent. DSRC offers good peer identification due to the smaller coverage area and selective admission of peer vehicles to the network [2].

Onboard units (OBUs) and Roadside units (RSUs) can be used together in connected vehicle applications to use DSRC communication. OBUs are devices that reside in vehicles and are used to send, receive, and forward information wirelessly through DSRC or other communication methods. RSUs are devices that are usually mounted in intersections, traffic infrastructure and the roadside. OBUs are easier to attach to and remove from CVs, whereas RSUs are usually fixed to the infrastructure and are less mobile.

5G and 4G-LTE cellular communication depend on the existing cellular wireless infrastructure, and offers low latency and high throughput simultaneously [3]. 5G and 4G-LTE can be redesigned to assist in vehicular cooperative communication and safety systems since they enable more bandwidth-demanding and real-time critical services.

5G and 4G-LTE communication-based systems have features that make them beneficial for automotive systems. They are energy efficient, provide better coverage, and have high down and uplink capacity. However, at higher vehicle speeds, they tend to have unreliable latencies in regions with high mobile network usage. Owing to the large coverage and centralized operation, identification of peers is often complicated and needs to be done onsite in the central server [2].

Cellular V2X (C-V2X, LTE-V2X) is a 3GPP standard for V2X applications and an alternative to DSRC. Abbasi et al. [4] focused on the comparison of C-V2X and DSRC communication for congested highway scenarios, deducting that both communication methods performed well for communication distances less than 100 m.

Recent developments in Unmanned Aerial Vehicle (UAVs) technology have brought about a wide range of areas where UAVs are or can be utilized for intelligent transportation system applications. The capabilities of a UAV can be expanded by introducing intervehicular communication. UAVs can be equipped with communication links to establish UAV-to-UAV or UAV-to-CAV communication, the latter being the focus of this paper. Dedicated Short Range Communication (DSRC) can be used to set up a communication link between UAVs and CAVs, for example.

## 2. Literature Review

Connectivity has been utilized widely for fuel economy improvement in CAVs in literature. Yu et al. [5] investigated fuel economy in the ecological driving of individual and platooning vehicles using longitudinal autonomy and connectivity. Altan et al. [6] modeled a V2I algorithm for longitudinal control of a CAV to get smooth acceleration and deceleration speed profiles using SPaT information from an upcoming traffic light. Sun et al. [7] investigated different fuel-optimal methods for speed planning of CAVs. Asadi and Vahidi [8] devised a V2I Model Predictive Controller (MPC) that uses upcoming traffic light information to obtain both fuel savings and a shorter trip time. Similarly, Yu et al. [9] designed an MPC for eco-driving; however, their focus was on a platoon rather than a single vehicle. Xu et al. [10] considered Eco-driving for transit rather than a personal vehicle to conserve fuel while reducing undesired emissions. Research has also been conducted on developing adaptive strategies for a dynamic roadway traffic environment while focusing on fuel savings [11]. Liu et al. [12] tested their vehicle following the control algorithm in a HIL setup with realistic V2X communication with a packet dropout compensator and have shown that their controller performed well even under non-ideal V2X communication.

Cantas et al. [13] and Kavas-Torris et al. [14] utilized DSRC communication for a Vehicle-to-Infrastructure (V2I) algorithm, so that Signal Phasing and Timing (SPaT) messages broadcast by a traffic light could be picked up by a CAV equipped with a DSRC modem to be used for fuel consumption reduction. Gelbal et al. [15] designed a Hardware-in-the-Loop (HIL) simulator to test automated driving algorithms and tested a Cooperative Adaptive Cruise Control (CACC) model utilizing DSRC communication for car following applications. Kavas-Torris demonstrated fuel economy improvement through V2I and V2V technologies for Eco-Driving of CAVs utilizing DSRC communication for MIL and HIL, as well as microscopic traffic co-simulation, implementation [16,17].

Simulation tools that enable researchers to test CAV-based algorithms are crucial for modelling and simulating algorithms that include connectivity [18]. Other than the small-scale implementation of connectivity technologies for a single CAV, the effects of having varying levels of CAV in a heterogeneous traffic flow have also been studied for future implementation of large-scale deployment of CAVs on public roadways [19,20].

Recent advancements in Unmanned Aerial Vehicle (UAVs) technology have brought about a wide range of areas where UAVs are utilized for intelligent transportation system applications. UAVs have been used in flight missions for search and rescue operations [21], as well as out-of-sight indoor flights with tactile feedback [22]. UAVs have also been utilized in the transportation of goods, parcels, and passengers [23]. In agriculture, UAVs can be used to monitor the height and health of crops using onboard cameras.

The capabilities of UAVs can be expanded by introducing inter-vehicular communication. UAVs can be equipped with communication links to establish UAV-to-UAV or UAV-to-CAV communication. Dedicated Short-Range Communication (DSRC) can be used to set up a communication link between UAVs and CAVs. Currently, Automatic Dependent Surveillance-Broadcast (ADS-B) is used as the standard protocol to transmit location information in the aerospace industry. However, Moore et al. [24] has suggested that ADS-B will not be able to handle low-altitude air traffic communication soon due to the expected increase in air traffic density at low altitude. Chakrabarty et al. [25] investigated how DSRC can be used as an alternative to ADS-B for UAV-to-UAV communication to prevent mid-air collisions at low altitudes. Menouar et al. [26] studied the applicability and challenges of how a UAV-enabled Intelligent Transportation System (ITS) for a smart city.

With the roll-out of the 6G networks, it will also be possible in the near future to have 6G-enabled UAV Traffic Management (UTM) ecosystems in dense and urban air-traffic scenarios, including aerial and satellite communication [27]. Khan et al. [28] focused on the integration of AI/ML with UAV networks utilizing the 6G ecosystem while keeping air interface and transmission technologies challenges of the 6G networks.

UAVs have also been conceptualized as a part of smart cities. Yilmaz and Denizer [29] explored how UAVs could be used in smart cities for traffic control. Hussain et al. [30] has focused on using UAVs for fire detection in smart city ecosystems and has expressed that their preliminary results have shown effective performance.

UAVs can also be utilized in coordinated missions with ground vehicles. Liu et al. [31] studied the joint scheduling of computation and communication resources in the collaborative networking of UAVs and platooning vehicles. Kavas-Torris et al. [32] developed use case scenarios to simulate CAV and UAV coordination, as well as demonstrated the hardware implementation for a DSRC based and a 4G WebSocket-based CAV and UAV communication link.

In this paper, hardware implementation and real-life testing of a coordinated CAV & UAV mission are presented. The Materials and Methods section starts by introducing details about the CAV platform and the UAV platform used for the hardware implementation. Then, the software platform used for the real-life testing, the Contingency Management Platform (CMP) that receives information from the CAV, as well as the companion onboard computer Raspberry Pi 4B and the Webserver that was used to display photos taken by the UAV during the flight operation, are presented. In the Hardware Implementation of UAV & CAV Communication section, the data flow between the CAV and UAV is explained, where DSRC, UDP, TCP, and WebSocket communications were used, respectively. In the Use Case Scenario Description section, the use case test scenario is described in detail. In the Test Results section, the success of the CAV and UAV communication is quantified through package drop percentage and latency analysis. In the Conclusion section, conclusions are drawn, and the next steps are elaborated.

## 3. Materials and Methods

In this section, each component of the CAV and UAV connectivity hardware implementation is explained. A CAV platform with DSRC connectivity was employed as the ground vehicle. For the aerial vehicle part, rather than choosing an off-the-shelf UAV, individual parts of the UAV were purchased and put together to get the UAV platform. A Raspberry Pi 4B was mounted on the UAV as a companion computer to handle image processing, connection to CMP and WebSocket servers, as well as data acquisition. In order to have an internet connection on the UAV, a 4G HAT was used with T-Mobile service provider to provide 4G internet to the Raspberry Pi 4B, as well as act as a Wi-Fi hotspot to nearby ground crews. A Logitech camera was mounted on the UAV to take pictures and survey the ground while the UAV was flying.

On the cloud side, the Contingency Management Platform (CMP) was used to get information from the CAV and UAV and to manage alerts for incidents.

### 3.1. Connected and Automated Vehicle (CAV) Platform

**Figure 1:**
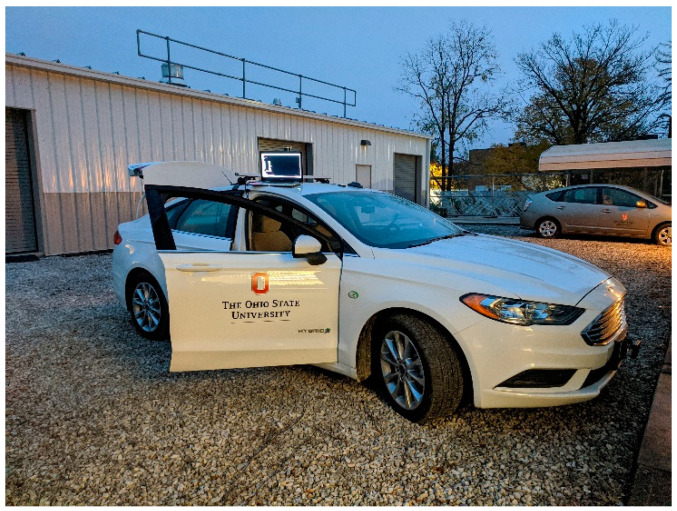
CAV platform Ford Fusion 2017 vehicle.

The CAV platform used for this manuscript can be seen below in Figure 1. The vehicle is a 2017 model Ford Fusion Hybrid vehicle with Drive-By-Wire. The CAV platform enables testing of V2V, V2X, and V2I algorithms, as well as Advanced Driver Assistance systems (ADAS). For this manuscript, the CAV platform was equipped with a DSRC modem to communicate with a flying UAV, which also was equipped with a DSRC modem.

### 3.2. Unmanned Aerial Vehicle (UAV) Platform

**Figure 2:**
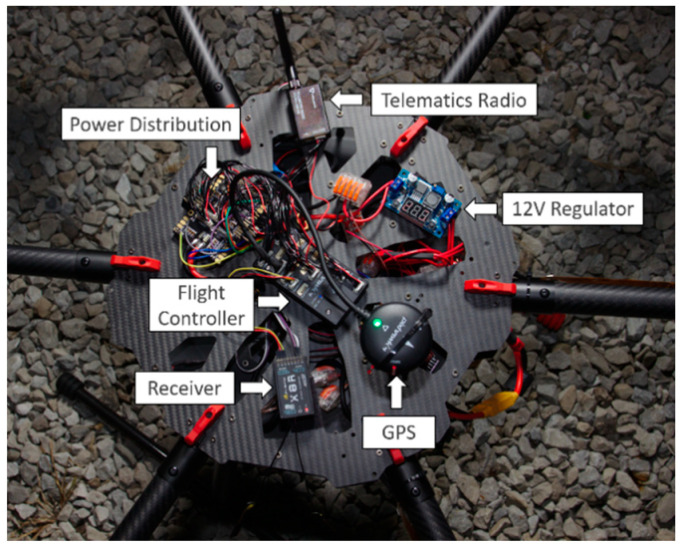
UAV platform.

The UAV platform used for this manuscript can be seen below in Figure 2. The UAV platform is a hexarotor with 6 rotors and has a 12 V voltage regulator. The UAV is equipped with a telematics radio, which enables it to be controlled by a pilot on the ground. The telematics unit also enables the UAV status to be monitored using a ground control station. For the work presented in this manuscript, the UAV was equipped with a DSRC modem to communicate with the CAV platform. For communication with other system elements, the Raspberry Pi 4B mounted on the UAV with 4G internet was utilized. Raspberry Pi 4B was also used to establish communication between the UAV and a WebSocket. Finally, a communication link was established between the UAV and the Contingency Management Platform (CMP).

### 3.3. Raspberry Pi 4B Companion Computer

Raspberry Pi 4B is a low-cost and energy-efficient electronic board mini computer that can be and has been, used for a variety of tech projects. Raspberry Pi 4B has a 1.5 GHz 64-bit quad-core Arm Cortex-A72 CPU, 8 GB RAM, integrated 802.11 ac/n wireless LAN, and Bluetooth 5.0 [33]. Additionally, it has 2 USB 2 ports, 2 USB 3 ports, and a gigabit ethernet port. The processing power of the Raspberry Pi 4B, as well as its compact size and low weight, makes it an ideal candidate for UAV research and flight operations.

For this study, the Raspberry Pi 4B was chosen as the companion computer and mounted on the UAV platform. It was connected to the camera for image processing. Additionally, the Raspberry Pi 4B was connected to the DSRC onboard-unit (OBU) modem on the UAV through the ethernet port for a UDP connection. The 4G HAT was connected to the Raspberry Pi 4B through USB to get 4G internet to the flying UAV. It was also used to establish a TCP port between itself and CMP.

### 3.4. 4G Internet

Even though DSRC communication is a reliable connection, an internet connection to transfer data from the UAV to a server might be necessary for real-life. For remote flights with no Wi-Fi access, equipping a UAV with an onboard 4G internet becomes crucial. For that reason, a 4G internet shield was added to the setup for an internet connection. By doing so, the WebSocket communication architecture was expanded for more realistic scenarios. For the 4G internet connection, a SIM7600A-H 4G HAT Board was used [34]. A SIM card with T-Mobile network provider was inserted to the card slot. On the software side, the necessary libraries and dependencies were resolved.

For this paper, due to the limitations of the hardware used during the experiments, a 4G network was used for the test. It should be noted that this internet connection can be upgraded to 5G and 6G in the future as they become available in the areas where the flight testing is conducted.

### 3.5. Logitech Camera and Image Processing

Using image processing, it is possible to extract useful information about roadways and vehicles travelling on roadways. CAVs and UAVs can benefit from this information in terms of fuel economy, ride comfort and mobility. Since information regarding traffic flow, average vehicle speed, presence of work zones, as well as queues and accidents can be detected using cameras in conjunction with UAVs, a companion computer setup with a camera was prepared.

For this implementation, a Logitech Webcam was connected through USB to the Raspberry Pi 4B. The small Logitech webcam had a 640 × 480 resolution. Python scripts were used along with the OpenCV library image processing tools. Additionally, for display purposes, OpenCV was used to capture frames. Then, the captured frames were sent from the onboard camera to the Webserver through Python scripts and WebSocket communication.

### 3.6. Websocket Server

WebSocket servers are applications that are programmed to listen to a TCP connection to ensure full-duplex communication. When WebSocket servers are being programmed, the first step is to make the server listen for incoming socket connections using a standard TCP socket. Then, the handshake must be established, where the details of the connection are negotiated. The WebSocket server also must keep track of the clients which have already completed the handshake. During the connection, either the server or the client can send messages at any time [35].

For this study, the client Raspberry Pi 4B first sent the handshake request to the server. When the connection was established between the WebSocket and the client, then the client Raspberry Pi sent photos taken by the Logitech camera to the WebSocket server. Once the WebSocket server received the photos, the messages and photos were displayed in a web browser. Other 3rd parties could also see the data received by the WebSocket server by going to the server address in their web browser and completing a handshake with the server as an observer.

### 3.7. Contingency Management Platform (CMP)

Contingency Management Platform (CMP) is a platform that can detect off-nominal conditions that could affect the UAV operations. Additionally, CMP can provide situational awareness and the impact of the off-nominal condition on UAVs. Expanding on that, it is possible to alert the UAVs to the existence of accidents when they are present. CMP can also be used to alert the UAV about several scenarios, one being flight zones to avoid for the UAV operations. For this real-life implementation, an accident location that was detected by the CAV was sent to the UAV, which then sent it to the CMP to alert all the UAVs connected to the CMP system.

## 4. Implementation of UAV & CAV Communication

For this paper, the following V2X communication methods were utilized for the implementation of UAV & CAV communication: DSRC, UDP, TCP, and WebSocket.

### 4.1. DSRC Communication between CAV & UAV

The Dedicated Short-Range Communication (DSRC) protocol has been utilized for communication needs in the automotive industry and has been used successfully for a wide variety of applications. Details and research work about DSRC were given in the Introduction section.

**Figure 3:**
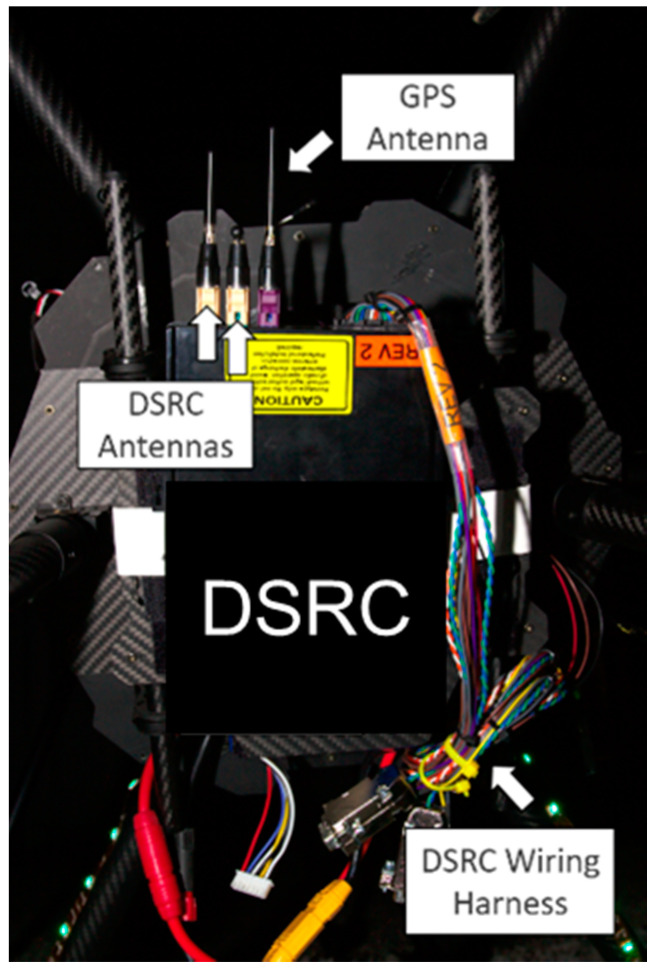
DSRC modem in the UAV platform.

UAVs can also be equipped with DSRC modems to send data to and receive data from DSRC-equipped aerial and ground vehicles. For this test, the CAV platform and the UAV platform were equipped with DENSO WSU 5900 DSRC modems. The modems and antenna configurations on the aerial and ground platform can be seen in Figure 3. Preliminary flight testing has been conducted to test the performance of the DSRC communication between a flying UAV and a stationary CAV and results have previously been published [32]. It has been shown that even though DSRC is a short-range communication protocol, it can be useful in low-altitude flight applications.

### 4.2. UDP Communication between DSRC Modem and Raspberry Pi 4B

The User Datagram Protocol (UDP) is an internet protocol suite that allows computer applications to send messages to other hosts on an Internet Protocol (IP) network. UDP uses a simple connectionless communication mode and provides checksums for data integrity with no error correction facilities. Using UDP, applications can use sockets to establish host-to-host communication. UDP connection prioritizes time over reliability, hence it is faster but less reliable, than TCP.

A UDP connection was established between the DSRC modem onboard-unit (OBU) and the Raspberry Pi 4B companion computer through the ethernet port. The Python library called “socket” was used to open a socket on the DSRC OBU side and receive the data from the open socket on the Raspberry Pi 4B side. Python lists were used to store latitude, longitude, and time information.

### 4.3. TCP Communication between Raspberry Pi 4B and CMP

The Transmission Control Protocol (TCP) is an internet protocol suite that provides reliable and error-checked delivery of a stream of bytes between applications. Since TCP is a connection-oriented protocol, a connection has to be established between the client and server before data can be sent. Having a TCP connection increases the latency in the data transfer, however, using TCP brings about a 3-way handshake and error detection.

Using the 4G internet on the UAV, a TCP connection was established between the Raspberry Pi 4B companion computer mounted on the UAV and the CMP. This link was used to send System and Fault Messages, which will be explained in Section 5.

### 4.4. Websocket Communication between Raspberry Pi and Webserver

WebSocket is a computer communication protocol that allows a two-way interactive communication between clients and a server over TCP. Using the WebSocket protocol, the WebSocket Server presented previously can interact with another web browser or other client applications. Therefore, it is possible to send messages and receive responses between one server and multiple clients.

Python scripting and WebSocket libraries [36] were used to design an 2-way WebSocket communication portal between the Raspberry Pi 4B and the Webserver through 4G internet. Frames captured from the live feed of the camera were sent to the Webserver and displayed in a web browser.

### 4.5. CAV and UAV Hardware Implementation

**Figure 4:**
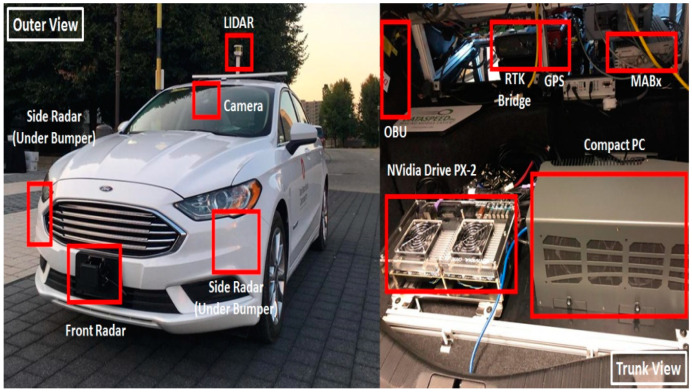
CAV platform equipped with hardware.

**Figure 5:**
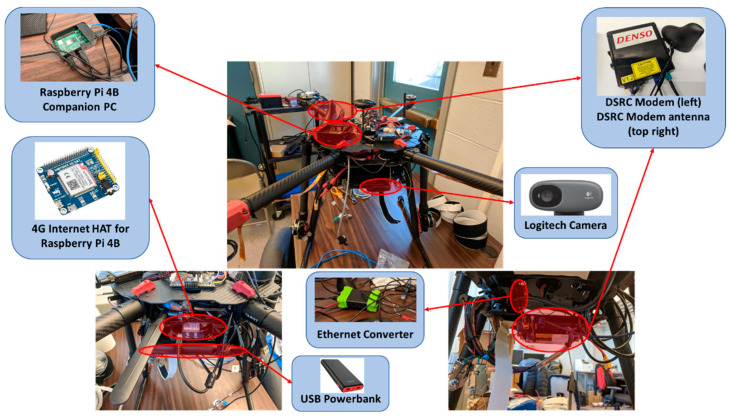
UAV platform equipped with hardware.

The CAV platform equipped with all hardware components can be seen in Figure 4. The UAV platform equipped with all hardware components can be seen in Figure 5.

## 5. Use Case Scenario Description

For the experiment, the parameters broadcast from the CAV and received by the UAV through DSRC communication are as follows:

- CAV latitude
- CAV longitude
- CAV altitude
- Message Transmit/Receive Timestamp
- GPS Time
- Remote Ground speed (m/s)

During the experiment, System Messages were sent from the UAV to CMP using TCP communication. The contents of the System Message are as follows:

- Message Transmit/Receive Timestamp
- CAV latitude
- CAV longitude
- CAV altitude
- UAV latitude
- UAV longitude
- UAV altitude

During the experiment, other than System Messages, Fault messages were also used to send information from the UAV to the CMP through TCP. The contents of the Fault Message are as follows:

- Incident latitude
- Incident longitude
- Incident altitude
- Incident time
- Incident type

For the use case, there was an active DSRC communication link between the CAV and the UAV, meaning that CAV and UAV shared location information with each other. The CAV location data was then transferred from the UAV OBU to the Raspberry Pi 4B companion PC that was mounted on the UAV through UDP communication. To establish communication between the Raspberry Pi 4B and the Contingency Management Platform (CMP), a TCP connection was set up, where a System Message was generated by the Raspberry Pi 4B. The System Message included information about CAV location (latitude, longitude, and altitude) and message transmit time stamp, as well as the UAV location (latitude, longitude, and altitude). The System Message was updated with live information from the CAV and UAV. When the CAV approached the predetermined accident/incident location, a Fault Message was generated by the Raspberry Pi 4B, sending the accident location to the CMP. Then, the accident location was displayed in the CMP. The accident location information was also used to call first responders to the accident location by the CMP command center staff. CMP also sent an alert to all air traffic around the accident location to land their UAVs. The UAV pilot, which was flying around the CAV and notified CMP of the accident, then landed the UAV.

**Figure 6:**
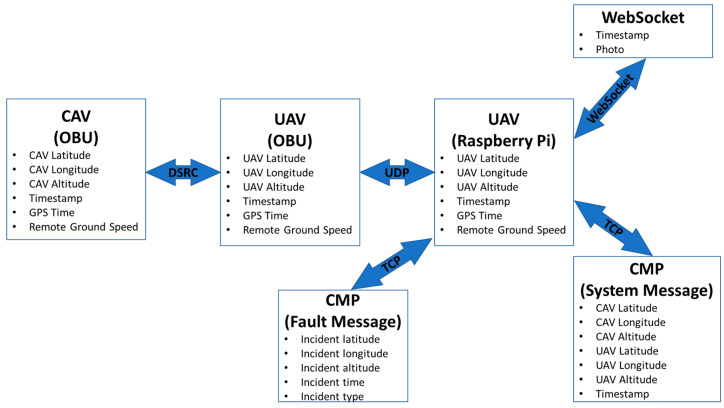
The messages sent and received between different platforms during the use case scenario.

In order to express the interaction between different platforms, the communication methods and message content, Figure 6 is given below.

**Figure 7:**
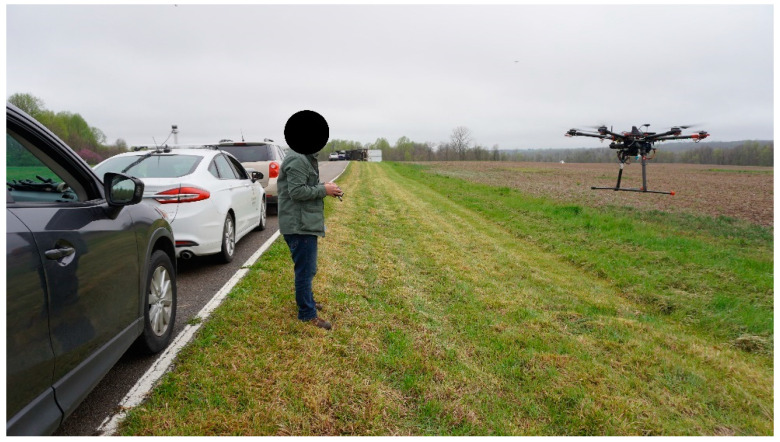
The field experiment with CAV and UAV.

In order to better visualize the experiment, an actual photo taken during the use case scenario Quick Clear demonstration can be seen below in Figure 7. In Figure 7, the white parked vehicle with the lidar on top is the CAV, and the flying UAV controlled by the pilot communicates with the CAV and CMP.

## 6. Test Results

To test the performance of the system, 2 experiments were run. In the following sections, the results of each experiment are explained in detail. While more performance metrics such as throughput and packet delivery ratio would also have been useful from a communication system performance analysis point of view, this approach is not taken here to make the presentation concise. This is also due to the fact that latency was important in our comparison of different ways of communicating data between a UAV and a CAV as we wanted to make sure that latency was small enough to use the chosen communication means in applications where this communication is needed. A comparison of data sent, and data received in the many experiments we conducted confirmed acceptable levels of performance.

### 6.1. Experiment #1 and Results

**Figure 8:**
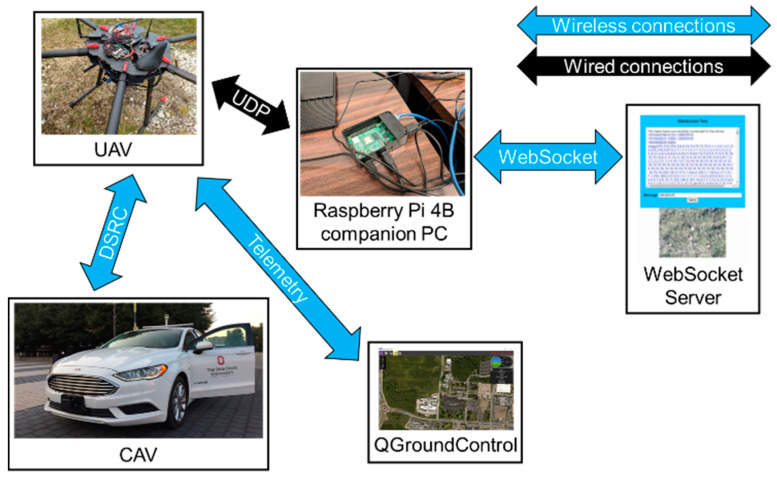
Schematic for experiment 1.

For the 1st experiment, DSRC communication was established between the CAV and UAV, so that location information could be shared between the ground and aerial vehicles. UAV telemetry was shared with another PC to monitor the health and state of the UAV by the pilot in QGroundControl. Then, DSRC data was passed to the Raspberry Pi 4B onboard companion PC using UDP to be further processed. Raspberry Pi 4B then established a connection through WebSocket with the WebSocket server, so that vehicle information, as well as photos taken with a camera mounted on the UAV, could be sent to the server to be displayed in a web browser. The schematic for the 1st experiment can be seen in Figure 8.

The main motivation of experiment 1 is to investigate the latency of different communication and computation methods. DSRC communication between the UAV and CAV corresponds to the direct reading of basic safety messages (BSM) of the CAV by the UAV and/or vice versa. The applications range from awareness to tracking of the nearby CAV(s) by the UAV for situational awareness of the traffic condition directly below the UAV. When it is needed, the UAV can also use its DSRC modem to send traffic guidance messages to the CAVs below much like a police officer directing traffic at an accident site. All the computational processing is taking place in the CAV and UAV DSRC modems in this case. If we want further processing of the information received from the CAV, the information from the CAV can be sent to the local computer, the Raspberry Pi board (or similar), for further local processing. The UDP latency shows the extra delay in transferring CAV data received through DSRC communication to the local UAV computational system for further processing. This process can also take place in the other direction when the local UAV computer sends data through UDP to its modem and through DSRC to the modem of the CAV. This is also a situation that occurs frequently in practice when the necessary computations cannot be handled by the UAV DSRC modem and the UAV local computer has to be used. Cellular modem (C-V2X) direct communication between the CAV and UAV could also have been used but was not used in this paper as we do not have C-V2X modems and as there are many vehicles (and also many roadside units) already equipped with DSRC modems in the area where we want to use this UAV-CAV communication in the future. The other method of communication that was tried is the web-socket server which uses 4G plus a cloud server to communicate indirectly and, interestingly, this mode of communication had the least latency meaning that this is a feasible method of communication between a CAV and a nearby UAV. Using a cloud server also means that information from the UAV including camera images can be sent to a centralized monitoring system for applications like accident site situational awareness, traffic monitoring, etc.

**Figure 9:**
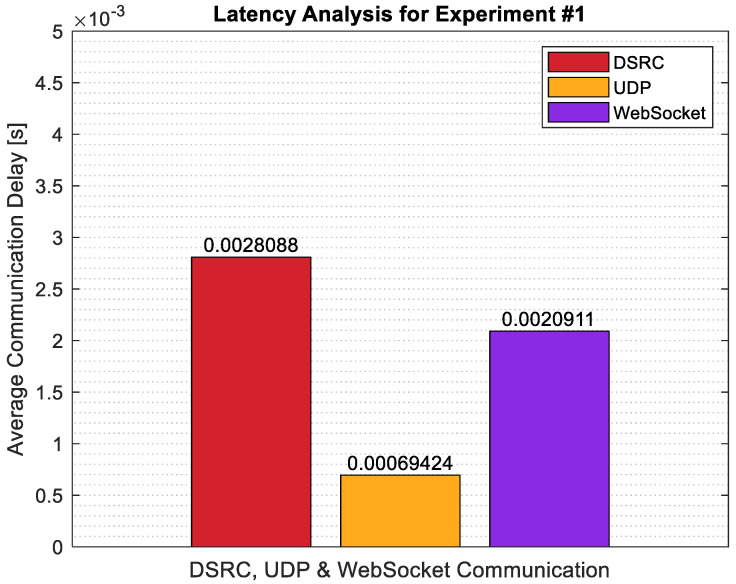
Latency analysis for Experiment #1.

**Figure 10:**
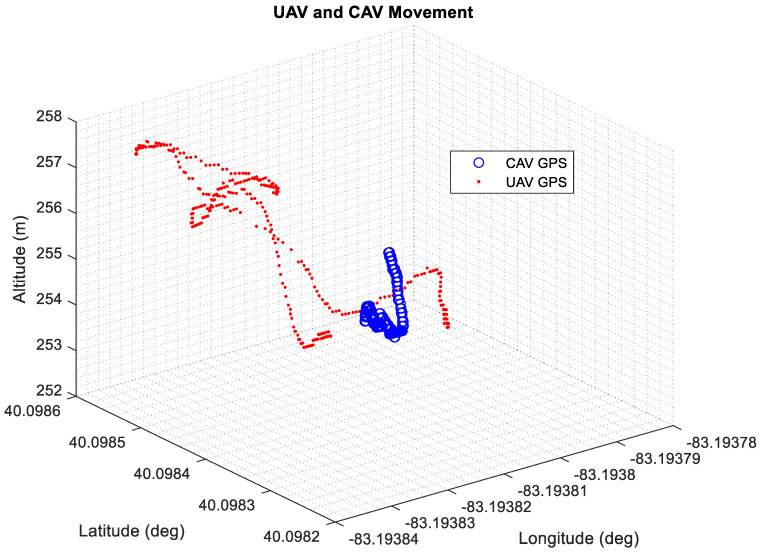
CAV GPS and UAV GPS locations for Experiment #1.

To capture the latency in the system, a detailed latency analysis was carried out for Experiment #1. Latency analysis was done for 3 different cases. For the 1st case, DSRC communication delay between the CAV and the UAV OBU’s was calculated, and the average was taken for the data recorded during Experiment #1. For the 2nd case, the UDP communication delay between the UAV OBU and the Raspberry Pi 4B was recorded and the average was taken. Lastly, the WebSocket communication delay between the photo being sent through the socket to the server and the server displaying the photo was recorded and the average was taken. The results are summarized in Figure 9. As seen in the figure, the smallest latency was observed for the UDP communication, followed by WebSocket. The slowest communication was observed for the DSRC communication. Other than the latency analysis, the GPS data from both the CAV and UAV were also plotted for this test. The CAV GPS location (in blue) and UAV GPS position can be seen in Figure 10. In the figure, the altitude of the CAV has an increasing trend for some data points due to the drift observed in the GPS.

### 6.2. Experiment #2 and Results

**Figure 11:**
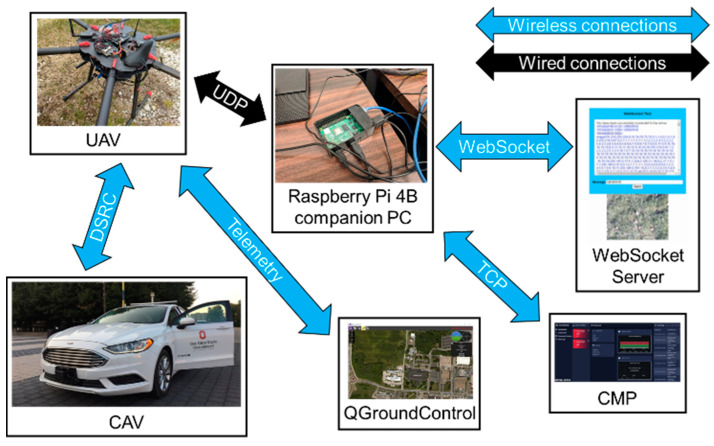
Schematic for Experiment #2.

The 2nd experiment builds up on the 1st experiment, such that the DSRC and WebSocket connections here are identical. However, in addition to the DSRC and WebSocket communication links in the 1st experiment, the Raspberry Pi 4B companion PC also established a TCP connection to the Contingency Management Platform (CMP) to send vehicle location information and if necessary, accident or incident location to CMP. The schematic for the 2nd experiment can be seen in Figure 11. Different hardware and software components were used in Experiment #2 to get the data transfer from the CAV OBU all the way to the Raspberry Pi 4B and the WebSocket server. The Raspberry Pi was monitoring each communication link during Experiment #2.

**Figure 12:**
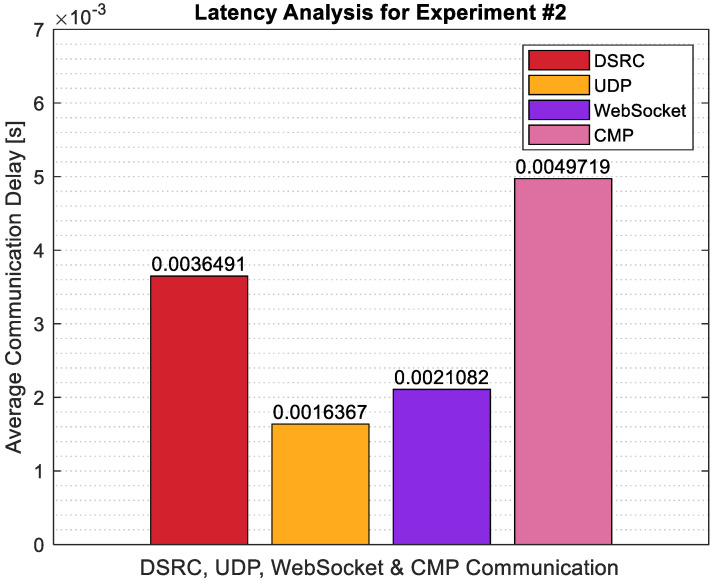
Latency analysis for Experiment #2.

To capture the latency in the system, a detailed latency analysis was carried out for Experiment #2. Latency analysis was done for 4 different cases. For the 1st case, DSRC communication delay between the CAV and the UAV OBU’s was calculated, and the average was taken for the data recorded during Experiment #2. For the 2nd case, the UDP communication delay between the UAV OBU and the Raspberry Pi 4B was recorded and the average was taken. For the third case, the WebSocket communication delay between the photo being sent through the socket to the server and the server displaying the photo was recorded and the average was taken. Lastly, the communication delay between Raspberry Pi 4B and CMP through the TCP connection was recorded, and the average was taken. The results are summarized in Figure 12. Looking at Figure 12, it is seen that similar to Experiment #1, the smallest latency was observed in UDP communication. The DSRC communication delay in Experiment #2 was larger than that of Experiment #1. The second smallest latency was observed in WebSocket communication and compared to Experiment #1, the latency was slightly larger in Experiment #2. CMP communication latency through the TCP port between the Raspberry Pi 4B companion computer and the CMP platform was the largest among all communication links.

**Figure 13:**
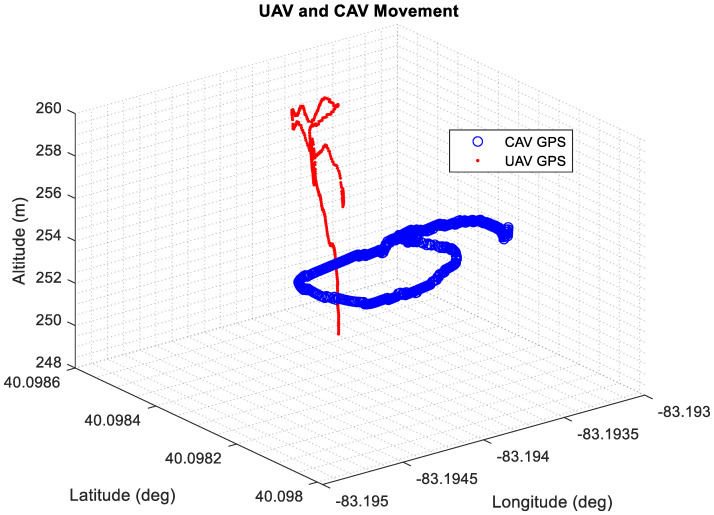
CAV GPS and UAV GPS locations for Experiment #2.

Another variation of this test was conducted, where whenever the UAV was flying around, CAV was also in motion and was operated by the driver to drive in a loop around the parking lot. The GPS data from the DSRC communication link between the CAV and UAV were plotted in a 3D plot and can be seen in Figure 13.

**Figure 14:**
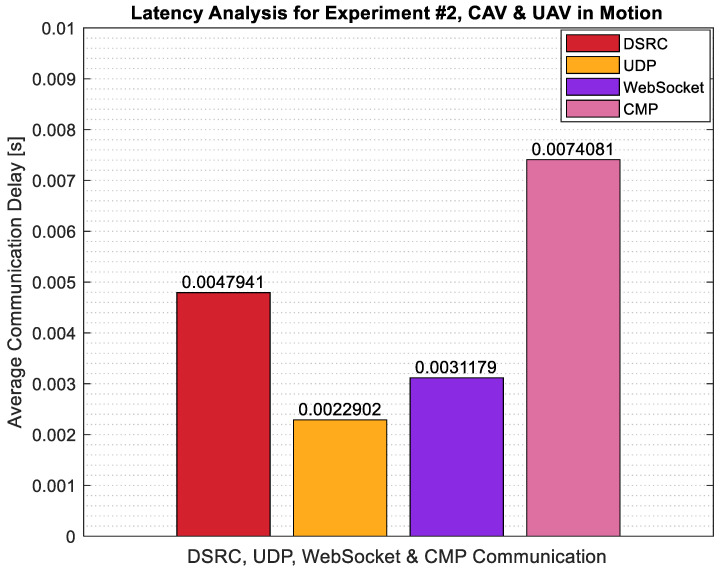
Latency analysis for Experiment #2, both CAV & UAV in motion.

For the second implementation of Experiment #2 with both CAV and UAV in motion simultaneously, the latency analysis was repeated. Looking at the results in Figure 14, it is seen that the latency in each communication link has increased compared to the version of Experiment #2 with only UAV in motion and CAV staying stationary. This result is as expected. During the experiment, the distance between the UAV and CAV changed more rapidly for this test and it resulted in a higher latency for the overall system.

Package drop percentage is another metric that can be investigated for the analysis of results. The data collected for all four communication methods, whose latency was quantified earlier, was also used to quantify the package drop percentage. Package drop percentage can be explained as the percentage of packages that were transmitted by the transmitter node of the communication, but not received on the receiver node side. The results for Experiment #2, for the case of both the CAV and UAV, were in motion during the test, are summarized in Table 1 for DSRC, UDP, WebSocket, and TCP communication. The DSRC communication between the CAV and the UAV had a package drop percentage of 0.36%. For the same experiment, the UDP communication between the UAV modem and Raspberry Pi 4B, the package drop percentage was found to be 1.72%. For the WebSocket communication between the Raspberry Pi 4B and the webserver, it was observed that the package drop percentage was 1.56%. Finally, for the TCP communication between the Raspberry Pi 4B and the CMP platform, the packet drop percentage was found to be 10.45%. The packet drop percentage values were calculated automatically by the modem or communication software used. These results show that out of these four communication methods, the DSRC had the least amount of package drop percentage which is expected as it is, just like its name, a dedicated short-range communication method.

## 7. Conclusions

In this paper, a V2X communication architecture was modeled and tested through real-life testing to show the potential of ground and aerial vehicle communication. Each system component was presented and explained in detail. Two test scenarios were devised, and those scenarios were tested through real-life testing. The results were analyzed, and special care was given to communication latency for the DSRC communication between the ground and aerial vehicle, UDP communication between the UAV OBU and Raspberry Pi 4B companion PC, WebSocket communication between the Raspberry Pi 4B companion computer and the WebSocket server, and the TCP communication between the Raspberry Pi 4B and CMP. After the latency analysis for Experiment #1, it was seen that the latency was minimal for each component, with DSRC having the least latency and UDP having the largest latency. For Experiment #2, however, it was observed that the UDP communication had a considerably large latency.

For future work, dedicated 4G LTE with priority from service towers could enhance the 4G internet connection, making the connection faster and more reliable, eliminating down time. The control of the CAV and UAV for coordinated and cooperative motion is also of significance for future work. Parameter space-based robust control [37,38] and model regulation [39,40] will be a good choice for controlling the path tracking of the CAV [41] and UAV as they have successfully been applied before in applications ranging from automotive control [42,43,44,45], friction compensation [46], robot force control [47] to atomic force microscope control [48] and collision avoidance [49].

In addition to using different control methods, more improvements can be made. In the field testing, the spot where the CAV was supposed to stop for the accident was predetermined. However, during the experiment, the actual GPS location of the CAV was sent to CMP in real-time. If the CAV does not know the accident location in real life, then further sensor information is required. Cameras, for instance, that are mounted on the CAV development platform can be used to detect when an accident occurs. Once the “accident flag” is triggered using the camera information, this information can be sent from the CAV to the UAV to be used further.

## Figures and Tables

**Table 1:** Summary of Package Drop Percentage comparison between the four communication methods utilized in Experiment #2.

Communication Methods	Package Drop Percentage (%)
DSRC	0.36
UDP	1.72
WebSocket	1.56
TCP	10.45

## Data Availability

Not applicable.
